# Children Show More Selective Cognitive Offloading After First Being Compelled to Offload Indiscriminately

**DOI:** 10.1111/cogs.70100

**Published:** 2025-08-05

**Authors:** Kristy L. Armitage, Alicia K. Jones, Jonathan Redshaw

**Affiliations:** ^1^ School of Psychology The University of Queensland

**Keywords:** Cognitive offloading, Strategy selection, Strategy perseveration, Cognitive development, Metacognition, Mental rotation, External normalization, Problem solving

## Abstract

With the rise of wearable technologies, mobile devices and artificial intelligence comes a growing pressure to understand downstream effects of cognitive offloading on children's future thinking and behavior. Here, we explored whether compelling children to use an indiscriminate cognitive offloading strategy affects their subsequent strategy selection. Six‐ to 9‐year‐olds (*N* = 128) completed a task where manual rotation of stimuli sometimes offloaded mental rotation demand and other times did not. In phase 1, some children were compelled to use manual rotation indiscriminately, whereas others could only use mental rotation. In phase 2, where children could freely choose their strategy, older children who were compelled to use manual rotation in phase 1 were significantly more selective in their strategy use, rotating the stimuli relatively more frequently when this behavior would offload cognitive demand than when it would not. These results provide preliminary evidence that pre‐exposure to indiscriminate cognitive offloading can promote selectivity in children's subsequent strategy use, though this selectivity may reflect a desire to avoid cognitive effort rather than improve task performance.

## 1 Introduction

Concerns regarding human reliance on external thinking tools are hardly new. In ancient Greece, Socrates worried that if people learned to write, it would “implant forgetfulness in their souls,” as internal memory processing would be replaced by “means of external marks.” These concerns continue to be echoed in modern‐day society, which is marked by unprecedented levels of opportunity for outsourcing cognitive operations. With the rise of wearable technologies, mobile devices and artificial intelligence comes a growing pressure to understand the downstream effects of *cognitive offloading* on thinking and behavior (Risko & Gilbert, 2016).

Over the past decade, systematic studies have observed that adults more frequently offload under conditions of higher cognitive demand (Dunn & Risko, 2015; Gilbert, 2015a; Risko & Dunn, 2015) and in response to relatively poor unaided performance (Gilbert, 2015b). These findings align with the metacognitive model of cognitive offloading, which posits that the decision to engage in or refrain from cognitive offloading is influenced by relevant metacognitive beliefs and experiences (Boldt & Gilbert, 2019; Hu, Luo, & Fleming, 2019; Risko & Gilbert, 2016). Indeed, adults often offload in line with their *beliefs* about task difficulty and their level of unaided competence, even when these beliefs fail to align with objective task demands (Dunn & Risko, 2015; Gilbert, 2015b). Adults’ cognitive offloading also appears to be strongly influenced by pre‐exposure to offloading strategies. In one study, participants who were required to use Google to answer difficult trivia questions were more likely to continue using Google when answering a new set of relatively easy trivia questions, compared to participants who were required to answer the initial questions from memory (Storm, Stone, & Benjamin, 2016). In another study, participants who were compelled to set reminders in the first half of a memory task were more likely to set reminders in the second half of the memory task when given a free choice, compared to those who were unable to set reminders in the first half of the task (Scarampi & Gilbert, 2020). While strategy perseveration in adults’ problem‐solving often reflects a preference for the strategy that affords the greatest benefit to performance (Weis & Kunde, 2024), it may also stem from a reluctance to expend extra cognitive resources on changing strategies from one trial to the next (Lemaire & Leclere, 2014). Alternatively, strategy perseveration may reflect a “mechanization in problem solving,” referred to as the *Einstellung effect*, where a strategy is repeatedly used even when it is unlikely to benefit performance, or when more appropriate or effective alternative strategies are available (Luchins, 1942).

It is now well‐established that, like adults, children are often inclined to offload cognitive demand (Armitage & Gilbert, 2025). In one study, 4‐ to 11‐year‐old children were given permission to manually rotate a turntable to eliminate the internal demands of mentally rotating the stimulus sheet on top of the turntable (Armitage, Bulley, & Redshaw, 2020). Critically, manual rotation was sometimes beneficial to performance and other times redundant. With increasing age, children were significantly more likely to manually rotate the turntable only when it would benefit them, demonstrating *selectivity* in their cognitive offloading decisions (see Armitage et al., 2020; Parrish et al., 2025; and Armitage, Li, Ng, & Redshaw, 2025; Bulley, McCarthy, Gilbert, Suddendorf, & Redshaw, 2020; Liang, Blaser, Yi, Sai, & Kaldy, 2025b; Liang, Kaldy, & Blaser, 2025a; Redshaw, Vandersee, Bulley, & Gilbert, 2018 for similar effects in mental rotation and memory tasks, respectively). Older children are also more likely than younger children to prospectively allocate external cognitive resources to trials with more internal cognitive demand (Dicken et al., 2025) and tailor their use of cognitive offloading strategies to their level of unaided competence (Armitage & Redshaw, 2022), potentially indicating increasing metacognitive awareness of situations in which offloading cognition would be most useful.

Recent work has also shown that with increasing age, children can effectively devise and deploy their own external cognitive strategies without any pre‐exposure to such strategies. For example, while completing a short‐term memory task, no 4‐ and 5‐year‐olds but the majority of 10‐ and 11‐year‐olds spontaneously devised their own external reminder‐setting strategies to improve their future recall (Bulley et al., 2020). Similarly, after experiencing an “impossible” memory task (see Armitage, Taylor, Suddendorf, & Redshaw, 2022), many children aged 6 years and older devised effective external strategies that were increasingly creative and flexibly deployed with age (Armitage et al., 2023). In some cases, however, children have refrained from spontaneously using offloading strategies that would have likely improved performance (as in Berry, Allen, Mon‐Williams, & Waterman, 2019); or have devised redundant external strategies apparently *aimed* at cognitive offloading that did not offload cognition at all (Armitage et al., 2020; Armitage & Redshaw, 2022).

While children's propensity to devise their own cognitive offloading strategies is now well understood, the downstream effects of previous experience with offloading strategies remain unknown. If children are shown how to routinely use an offloading strategy in a particular context, then might they simply continue to use this strategy indiscriminately even when they derive no benefit from it? One might wonder, for instance, whether children who are taught to use a calculator would become inclined to over‐use it to solve basic arithmetic problems (e.g., 2 + 2) instead of exercising their own internal abilities for arithmetic. Broadly, it is possible that becoming accustomed to such offloading strategies may interfere with children's experience with and aptitude for making accurate metacognitive evaluations about the usefulness of such strategies in various conditions. With the ever‐growing number of offloading opportunities available at children's fingertips, and concerns about the cognitive consequences of excessive offloading (Armitage & Gilbert, 2025; Carr, 2008; Grinschgl, Papenmeier, & Meyerhoff, 2021), it is increasingly important to clarify the optimal level of exposure to offloading strategies that will enable modern‐day children to be effective cognitive agents in the digital age.

In the current study, 6‐ to 9‐year‐old children participated in an adapted version of Armitage et al.'s (2020) mental rotation task, where they were presented with stimulus sheets on top of a rotatable turntable. Each stimulus sheet showed several stick figures that varied on two dimensions: (1) they either had red or blue faces, and (2) either had their arms pointed up or down. Children were either asked to count the figures with blue faces or the figures with their arms pointed up. While counting the figures’ arms was more cognitively demanding in the inverted than the upright orientation, counting the figures’ color was equally easy in both orientations because of a visual pop‐out effect. In other words, using the turntable to manually rotate the stimulus sheets to the upright orientation served to offload cognition on the arms but not color trials. Critically, in phase 1, children were either compelled to engage in *external normalization* (see Risko, Medimorec, Chisholm, & Kingstone, 2014) by manually rotating stimulus sheets to the upright orientation on all trials, even when it served no benefit to performance (rotator condition), or on none of the trials (non‐rotator condition). In phase 2, we examined whether children persevered with their allocated phase 1 strategy when given a free choice, or whether they offloaded selectively (i.e., used external normalization on arms trials more than color trials, as in Armitage et al., 2020).

For phase 1, we predicted that children in the rotator condition would provide more accurate answers than those in the non‐rotator condition on trials where external normalization offloaded mental rotation demand (i.e., arms trials), but not on trials where external normalization was redundant (i.e., color trials). Given the novelty of the research question, we did not make directional hypotheses about the influence of pre‐exposure to external normalization on children's cognitive offloading decisions but preregistered our intention to explore condition‐level differences in children's cognitive offloading when given a free choice in phase 2, and whether any such effects varied with age.

## 2 Method

### 2.1 Participants

This experiment was preregistered on the Open Science Framework (https://osf.io/9ghrv) and received ethical approval (Clearance ID: 2019/HE000267) prior to data collection. The final sample was 128 children (64 male, 64 female) aged between 6.04 and 9.99 years (*M* = 7.99, *SD* = 1.16), as preregistered, which was made up of 32 6‐year‐olds (*M* = 6.49, *SD* = 0.30), 32 7‐year‐olds (*M* = 7.51, *SD* = 0.25), 32 8‐year‐olds (*M* = 8.46, *SD* = 0.28), and 32 9‐year‐olds (*M* = 9.50, *SD* = 0.36). An additional five children were excluded due to experimenter error (*n* = 3) and being unable or unwilling to engage with (*n* = 1) or complete the task (*n* = 1). The sample was mostly White and middle‐class, recruited from a public museum with free entry in a medium‐sized and industrialized Australian city. All children spoke fluent English and had no clinical diagnoses at the time of testing. Participation was voluntary, and both written caregiver consent and verbal child assent was obtained before the experiment began.

### 2.2 Procedure

**Fig. 1:**
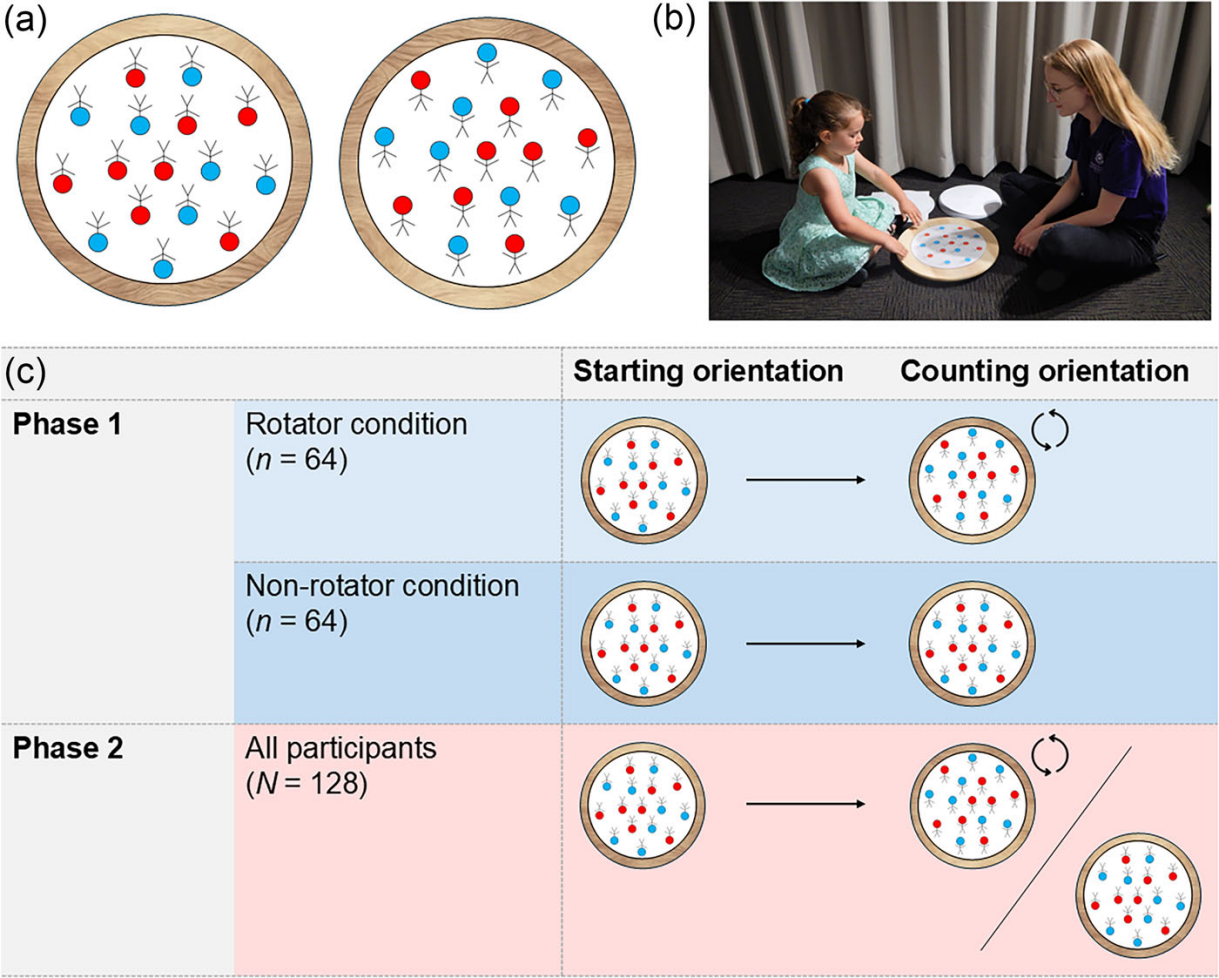
Panel A shows a visual representation of a stimulus sheet in the inverted orientation (left side) and the upright orientation (right side). The orientation of the stimulus sheet makes no difference to counting the number of figures with blue faces, but the figures with their arms up are easier to count in the upright than the inverted orientation. Panel B shows an image of a child completing the task with an experimenter. Panel C shows the condition manipulation in phase 1, where children in the rotator condition were required to rotate the stimulus sheets to the upright orientation before counting on every trial, whereas children in the non‐rotator condition were prohibited from manually rotating the stimulus sheets on any trial. All children were given a free choice on every trial in phase 2.

Children sat opposite the experimenter, with a single rotatable turntable placed on the ground between them (see Fig. 1, Panel B). After completing a preliminary activity demonstrating that the turntable could rotate (see S1 in the Supplementary Materials), children were introduced to an adapted version of Armitage et al.'s (2020) task. On each trial, the experimenter placed a stimulus sheet on the turntable, showing 16 stick figures that varied on two dimensions: (1) they either had a blue or red face, and (2) they either had their arms pointed up or down (see Fig. 1, Panel A). All sheets were presented in the inverted orientation (180 degrees), and children were asked to count either the number of blue people (color trials) or the number of people with their arms pointed up (arms trials). Critically, in this orientation, counting the number of blue people is less cognitively demanding than counting the number of people with their arms pointed up, as color visually “pops out” in perception (see Armitage et al., 2020; Treisman & Gelade, 1980).

In phase 1, children were pseudorandomly assigned to an experimental condition, with the constraint that an equal number of males and females per age completed each condition. Children assigned to the *rotator* condition were instructed by the experimenter to manually rotate the figures to the upright orientation prior to counting on all phase 1 trials, regardless of whether they were counting color or arms. Children assigned to the *non‐rotator* condition, by contrast, were prohibited from manually rotating the figures on any phase 1 trial, such that all figures were counted in the inverted orientation (see Fig. 1, Panel C). In phase 2, children in both conditions were able to choose whether to rotate the turntable, with the experimenter saying “Whenever you want to move it, you can. But you don't have to – it's your choice.” To account for issues with perceived permission, children also received reminders after every second phase 2 trial: “Remember, you can move it if you want to, but you don't have to” (see S2 in the Supplementary Materials for the experimenter script). Children completed eight trials (four color and four arms trials) in each phase in a counterbalanced order (see S3 in the Supplementary Materials for full counterbalancing information).

## 3 Results

### 3.1 Analysis plan

All data were analyzed using generalized linear mixed models (GLMMs; for models with binary dependent variables) or linear mixed models (LMMs; for models with continuous dependent variables) with a random intercept for participant using the statistical program SAS 9.4 (SAS Institute, 2013). Binary dependent variables were modeled using a binomial distribution and a logit link function. As specified in our preregistration, age was analyzed as a continuous mean‐centered variable to capture linear developmental changes, and significant age effects were followed up by splitting the sample into younger and older age groups: (1) 6‐ and 7‐year‐olds (*N* = 64, *M* = 7.00, *SD* = 0.58) and (2) 8‐ and 9‐year‐olds (*N* = 64, *M* = 8.98, *SD* = 0.62).

For each dependent variable of interest, we ran an initial main effects model and then added interaction terms into separate follow‐up models. Each interaction model, which included all relevant main effects and the added interaction term, was compared to the initial main effects model using Likelihood Ratio Tests. Effect sizes (*w*; Cohen, 1992) are reported throughout, and Bonferroni corrections have been applied to *p*‐values for follow‐up tests involving multiple comparisons (i.e., by multiplying the obtained *p*‐value by the number of comparisons and capping the result at > .999). Any exploratory analyses are clearly labeled. Full model details (including summary tables for each analysis and Likelihood Ratio Tests) can be found in S4−S8 of the Supplementary Materials.

#### 3.1.1 Assumption checking

Inspection of all initial main effects models indicated that random intercepts were approximately normally distributed for each dependent variable, except for phase 2 accuracy and phase 2 time‐on‐task, which had positively skewed intercepts. We, therefore, analyzed these variables using marginal models (generalized estimating equations; GEEs) which, unlike conditional models (GLMMs, LMMs), require no assumption of normally distributed random effects (Muff, Held, & Keller, 2016; see S7 and S8 in the Supplementary Materials for the GEEs).

### 3.2 Phase 1 accuracy (preregistered)

**Fig. 2:**
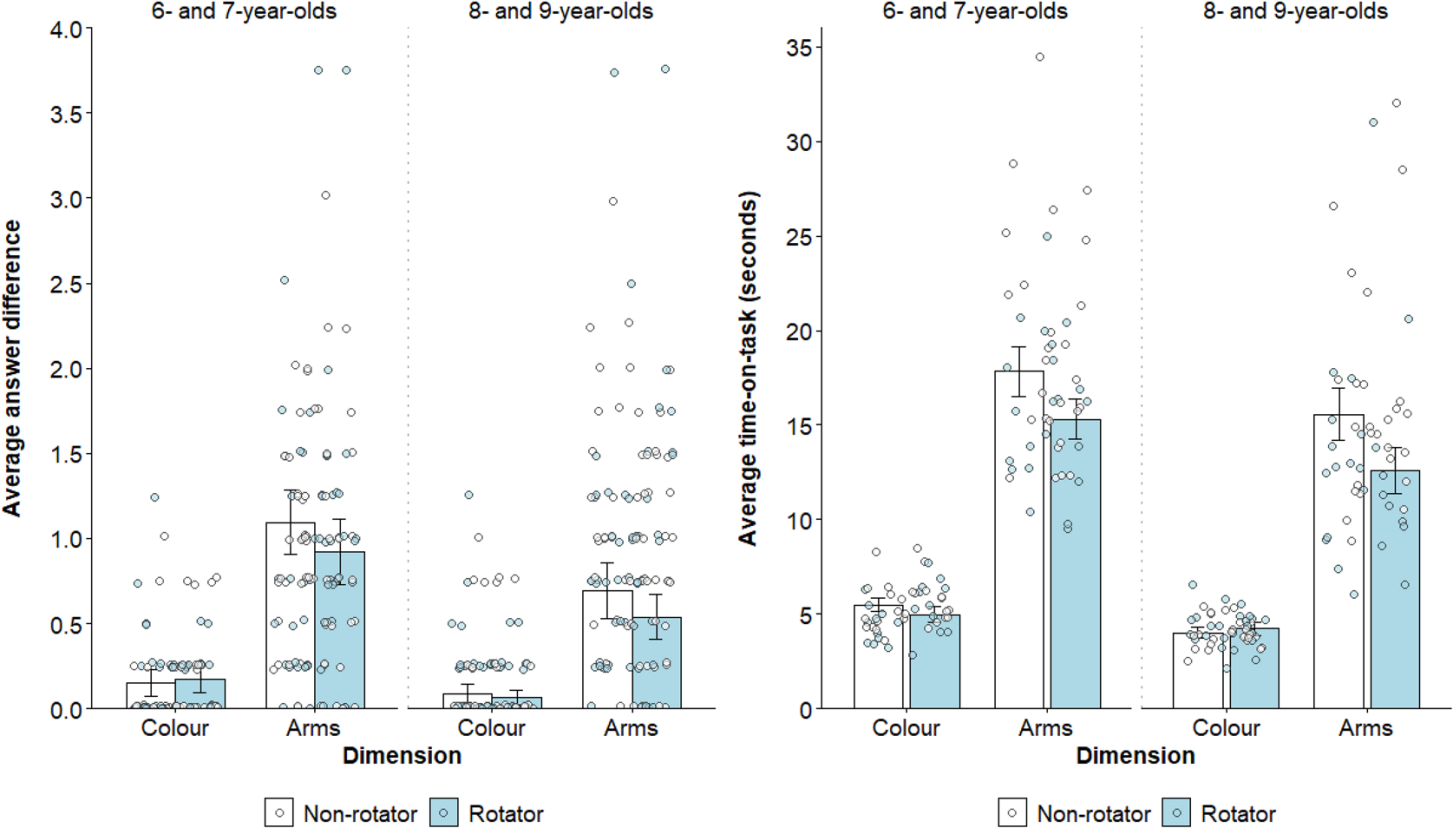
**Left**: Children's average phase 1 accuracy (continuous, difference between given answer and correct answer) in each condition (rotator and non‐rotator), split according to dimension (color and arms) and age group. Individual averages are represented by circles, color‐coded according to condition. Error bars represent 95% confidence intervals. **Right**: Children's average phase 1 time‐on‐task (continuous, in seconds) in each condition, split according to dimension and age group. Individual averages are represented by circles, color‐coded according to condition. Error bars represent 95% confidence intervals. All figures were made using the ggplot2 package (v3.5.1; Wickham, 2016) in RStudio (R Core Team, 2024).

Phase 1 counting accuracy (continuous; absolute difference between correct answer and given answer) was modeled as a function of age (continuous; mean‐centered), condition (rotator or non‐rotator), dimension (color or arms), and trial number per dimension (continuous; 1–4), controlling for sex (male or female) and counterbalancing order (1–8). The significant main effects of age, *χ* ^2^(1, 893) = 20.21, *p* < .001, *w* = 0.40, and dimension, *χ* ^2^(1, 893) = 250.66, *p* < .001, *w* = 1.40, indicated that children provided more accurate responses with increasing age (b = −0.13), and on color trials (*M* = 0.12) compared to arms trials (*M* = 0.85; b = 0.73; see Fig. 2). A significant interaction between the two variables, *χ* ^2^(1, 892) = 21.31, *p* < .001, *w* = 0.41, indicated that the effect of dimension was less pronounced for older children, *χ* ^2^(1, 893) = 74.44, *p* < .001, *w* = 1.08 (b = −0.56), compared to younger children, *χ* ^2^(1, 893) = 194.26, *p* < .001, *w* = 1.74 (b = −0.90). The three‐way interaction between age, condition, and dimension was also significant, *χ* ^2^(3, 891) = 21.32, *p* < .001, *w* = 0.41, but follow‐up analyses indicated no significant differences in response accuracy on arms trials, *χ* ^2^s < 2.72, *p*s > .397, *w*s < 0.21, or color trials, *χ* ^2^s < 0.06, *p*s > .999, *w*s < 0.03, across conditions in either age group (see Fig. 2). In other words, children in the rotator condition were not significantly more accurate when counting arms than children in the non‐rotator condition, inconsistent with the assumption that counting arms was easier in the upright than inverted orientation (as in Armitage et al., 2020). We clarify these unexpected effects below (see Phase 1 Time‐on‐Task). All other main effects, *χ* ^2^s < 2.07, *p*s > .150, *w*s < 0.13, and the preregistered interaction between condition and dimension, *χ* ^2^(1, 892) = 2.88, *p* = .090, *w* = 0.15, were nonsignificant. Note that this pattern of results replicates when treating phase 1 accuracy as a binomial dependent variable (correct or incorrect; see S9 in the Supplementary Materials).

### 3.3 Phase 1 time‐on‐task (exploratory)

Unexpectedly, the accuracy results from phase 1 were not consistent with the assumption that manual rotation improves accuracy on arms trials, but not on color trials (as in Armitage et al., 2020), which can likely be explained by a slight variation in experimental instructions. In Armitage et al. (2020), children were instructed to count as fast as possible, whereas in our instructions, we did not emphasize the importance of speed. Indeed, it is possible, although time‐consuming (Estes, 1998; Shepard & Metzler, 1971), to answer accurately on arms trials using mental rotation alone, and we suspect that many children in the non‐rotator condition were striving for perfect accuracy due to the absence of any time pressure. We, therefore, considered that the offloading benefit afforded by manual rotation may be better quantified by comparing children's *time‐on‐task* across conditions. To explore this possibility, we coded time‐on‐task using video footage of testing sessions, which was available for 77% of participants. Individual trials on which time‐on‐task was above or below 3SDs from the overall means for each dimension were excluded (14 phase 1 trials and 12 phase 2 trials). A second rater independently coded phase 1 time‐on‐task for 20% of the available sample, with inter‐rater reliability analyses indicating excellent agreement (*r* = .99, *p* < .001).

Phase 1 time‐on‐task was measured either from the moment the stimulus sheet was placed on the turntable (for non‐rotators) or was rotated to the upright orientation (for rotators) to the moment the child provided an answer (to reflect the period that children should have been counting in each condition). Phase 1 time‐on‐task (continuous, mean‐centered) was modeled as a function of age, condition, dimension, and trial number, controlling for sex and counterbalancing order. All focal main effects were significant, *χ* ^2^s > 6.89, *p*s < .009, *w*s > 0.23, indicating that children spent less time‐on‐task with increasing age (b = −0.67), in the rotator condition (*M* = 9.42 s) compared to the non‐rotator condition (*M* = 11.21 s; b = 1.73), on color (*M* = 4.69 s) compared to arms trials (*M* = 16.01 s; b = −11.36), and on later compared to earlier trials (b = −0.78). A significant two‐way interaction between dimension and condition, *χ* ^2^(1, 671) = 24.76, *p* < .001, *w* = 0.44, indicated that, as suspected, children in the rotator condition spent less time‐on‐task than those in the non‐rotator condition on arms trials, *χ* ^2^(1, 671) = 25.01, *p* < .001, *w* = 0.44 (b = 3.40), with no difference between conditions on color trials, *χ* ^2^(1, 671) = 0.01, *p* > .999, *w* = 0.01 (b = 0.06; see Fig. 2). In other words, consistent with well‐established mental rotation effects, counting arms in the inverted orientation was more time‐consuming (and thus cognitively effortful; Estes, 1998; Shepard & Metzler, 1971) than counting arms in the upright orientation, indicating that manual rotation on arms trials did indeed serve to offload cognitive demand.

### 3.4 Phase 2 rotation (preregistered)

**Fig. 3:**
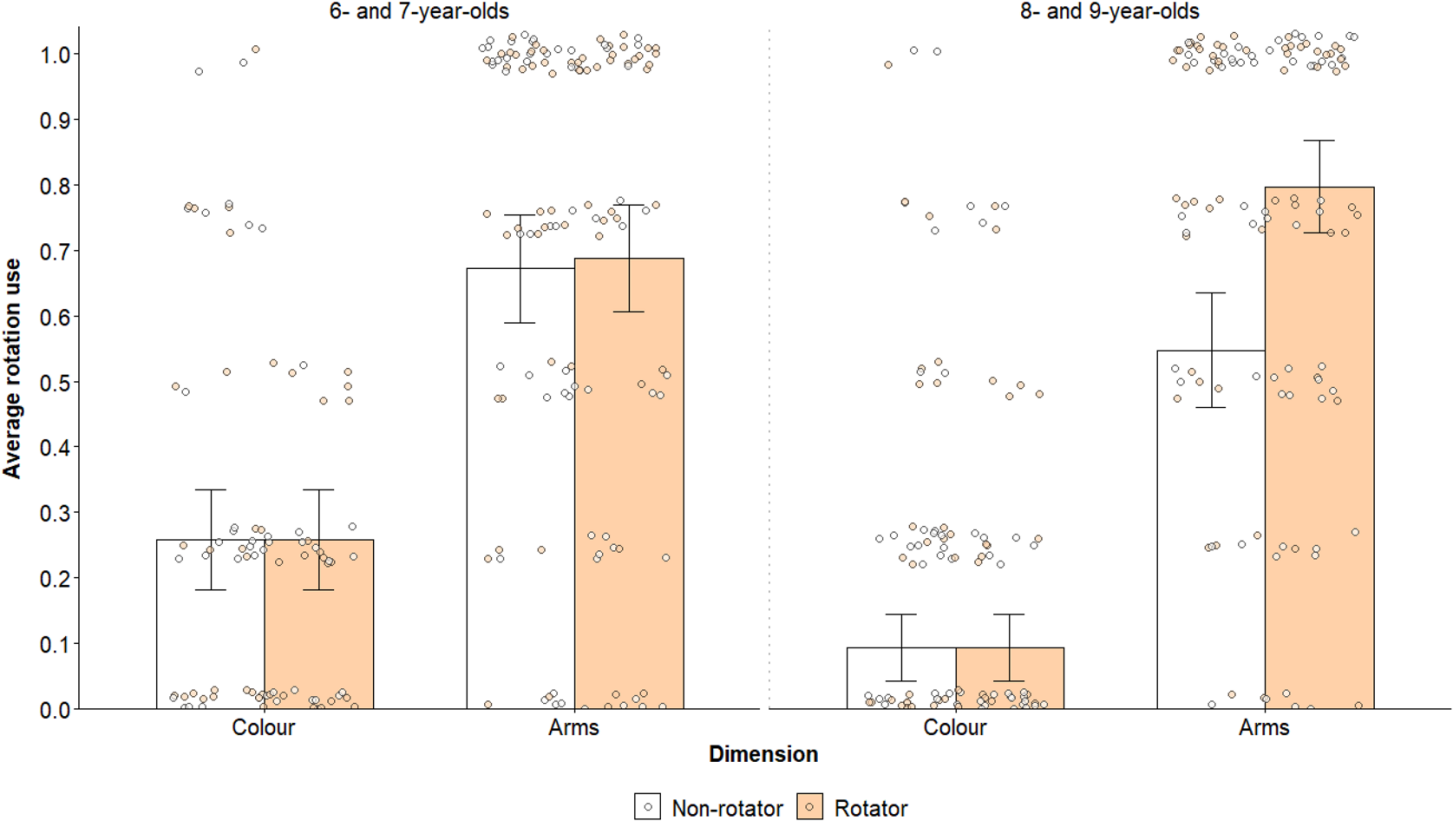
Children's average use of manual rotation (continuous; 0–1) in each condition (non‐rotator and rotator), split according to counting dimension (color and arms) and age group. Individual averages are represented by circles, color‐coded according to condition. Error bars represent 95% confidence intervals. All figures were made using the ggplot2 package (v3.5.1; Wickham, 2016) in RStudio (R Core Team, 2024).

Phase 2 rotation (binary; rotation or no rotation) was modeled as a function of age, condition, dimension, and trial number, controlling for sex, counterbalancing order, and whether trials were immediately preceded by a rotation reminder (binary, yes or no; see the Procedure section above). A significant main effect of dimension, χ^2^(1, 892) = 210.07, *p* < .001, *w* = 1.28 (b = 3.57), indicated that children rotated more frequently on arms trials (*M* = 0.68) than color trials (*M* = 0.18), confirming that children overall offloaded selectively (see Fig. 3). Consistent with Armitage et al. (2020), the dimension effect significantly interacted with age, *χ* ^2^(1, 892) = 22.69, *p* < .001, *w* = 0.42, revealing that older children, *χ* ^2^(1, 892) = 137.15, *p* < .001, *w* = 1.46 (b = −4.51), were more selective than younger children, *χ* ^2^(1, 892) = 98.85, *p* < .001, *w* = 1.24 (b = −2.86). The effect of dimension also significantly varied by trial, *χ* ^2^(1, 892) = 7.23, *p* = .007, *w* = 0.24. Follow‐up analyses suggested that children's use of rotation tended to increase across trials when counting arms, *χ* ^2^(1, 892) = 5.00, *p* = .051, *w* = 0.20 (b = 0.26), and decrease across trials when counting color, *χ* ^2^(1, 892) = 2.03, *p* = .308, *w* = 0.13 (b = −0.19), although neither effect met the conventional threshold for significance.

Critically, the three‐way interaction between age, condition, and dimension was also significant, *χ* ^2^(3, 890) = 26.20, *p* < .001, *w* = 0.45. Follow‐up analyses revealed that younger children's use of rotation did not vary by condition on either color, *χ* ^2^(1, 890) = 0.15, *p* > .999, *w* = 0.05 (b = 0.24), or arms trials, *χ* ^2^(1, 890) < 0.01, *p* > .999, *w* < 0.01 (b < 0.01). Older children's use of rotation also did not vary by condition when counting color, *χ* ^2^(1, 890) = 0.01, *p* > .999, *w* = 0.01 (b = −0.07), but those in the rotator condition offloaded significantly more frequently than those in the non‐rotator condition when counting arms, *χ* ^2^(1, 890) = 10.45, *p* = .005, *w* = 0.40 (b = 2.04; see Fig. 3). This finding indicates that, among older children, participants who were compelled to rotate in phase 1 were more selective in their offloading strategies in phase 2. All other main effects, *χ* ^2^s < 3.82, *p*s > .051, *w*s < 0.17, and interactions, *χ* ^2^s < 3.67, *p*s > .160, *w*s < 0.17, were nonsignificant. See S10 in the Supplementary Material for exploratory analyses examining the effect of phase 1 accuracy on children's rotation behaviors.

### 3.5 Phase 2 accuracy (preregistered)

**Fig. 4:**
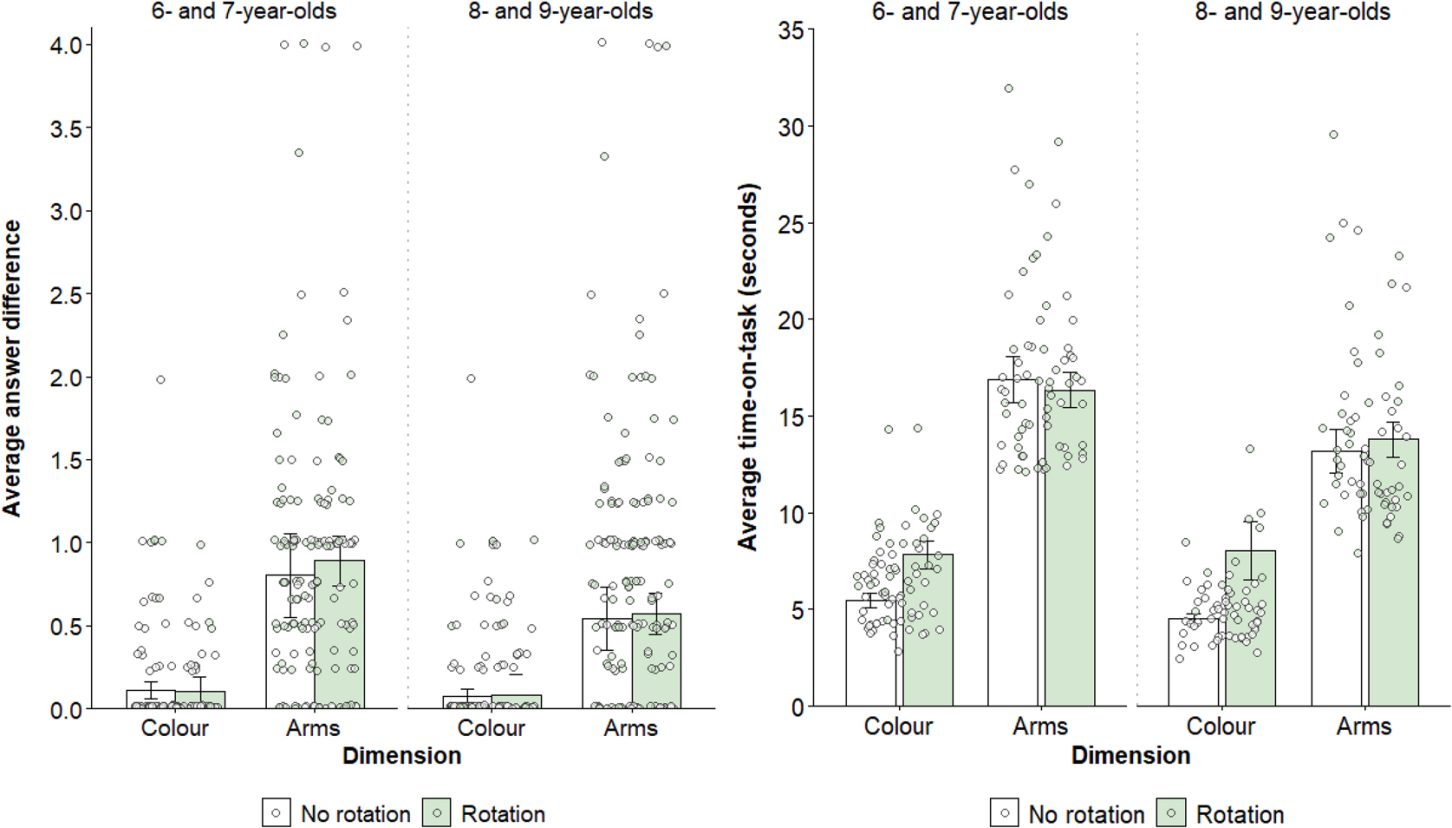
**Left**: Children's average phase 2 accuracy (continuous, difference between given answer and correct answer) on trials with and without rotation, split according to dimension (color and arms) and age group. Individual averages are represented by circles, color‐coded according to rotation. Error bars represent 95% confidence intervals. **Right**: Children's average phase 2 time‐on‐task (continuous, in seconds) on trials with and without rotation, split according to dimension and age group. Individual averages are represented by circles, color‐coded according to rotation. Error bars represent 95% confidence intervals. All figures were made using the ggplot2 package (v3.5.1; Wickham, 2016) in RStudio (R Core Team, 2024).

Phase 2 counting accuracy (continuous; absolute difference between correct answer and given answer) was modeled as a function of age, condition, dimension, use of rotation (binary; rotation or no rotation), and trial, controlling for sex and counterbalancing order. The significant main effects of age, *χ* ^2^(1) = 12.72, *p* < .001, *w* = 0.32, and dimension, *χ* ^2^(1) = 42.20, *p* < .001, *w* = 0.57 (b = 0.63), indicated that children were more likely to provide correct answers with increasing age, and on color trials (*M* = 0.09) compared to arms trials (*M* = 0.73; b = −0.10; see Fig. 4). All other main effects, *χ* ^2^s < 0.97, *p*s > .324, *w*s < 0.09, were nonsignificant. The interaction between dimension and use of rotation was also nonsignificant, *χ* ^2^(1) = 1.06, *p* = .302, *w* = 0.09, but was qualified by a significant exploratory three‐way interaction between age, dimension, and use of rotation, *χ* ^2^(3) = 11.51, *p* = .009, *w* = 0.30. However, follow‐up analyses indicated no significant differences in response accuracy as a function of rotation on arms or color trials for either age group, *χ* ^2^s < 0.37, *p*s > .999, *w*s < 0.08 (see Fig. 4). We clarify these unexpected effects below (see Phase 2 Time‐on‐Task). Note that this pattern of results replicates when treating phase 2 accuracy as a binomial dependent variable (correct or incorrect; see S9 in the Supplementary Materials).

### 3.6 Phase 2 time‐on‐task (exploratory)

In phase 2, time‐on‐task was measured from the moment the stimulus sheet was placed on the turntable to the moment children provided an answer (given that all children had a choice to rotate or not). A second rater independently coded phase 2 time‐on‐task for 20% of the available sample, with inter‐rater reliability analyses indicating excellent agreement (*r* = .99, *p* < .001). Phase 2 time‐on‐task (continuous, mean‐centered) was modeled as a function of age, condition, dimension, trial, and rotation, controlling for sex and counterbalancing order. Children spent less time‐on‐task with increasing age, *χ* ^2^(1) = 10.52, *p* = .001, *w* = 0.29 (b = −0.75), and on color (*M* = 5.45 s) compared to arms (*M* = 15.06 s) trials, *χ* ^2^(1) = 62.26, *p* < .001, *w* = 0.70 (b = 9.13; see Fig. 4). All other main effects were nonsignificant, *χ* ^2^s < 2.93, *p*s > .087, *w*s < 0.15.

A significant interaction between dimension and rotation, *χ* ^2^(1) = 6.56, *p* = .010, *w* = 0.23, indicated that children spent more time‐on‐task on trials with rotation when counting color, *χ* ^2^(1) = 27.34, *p* < .001, *w* = 0.46 (b = 2.47; see Fig. 4). Given that color was similarly easy to count in both orientations—as shown in the phase 1 time‐on‐task analyses—this difference is likely attributable to the additional time spent rotating the turntable (during which children would not be expected to count). Indeed, when counting arms, children showed no significant difference in overall time‐on‐task on trials with and without rotation, *χ* ^2^(1) < 0.01, *p* > .999, *w* < 0.01, (b = −0.03; see Fig. 4), which likely reflects that counting arms was easier in the upright orientation and yet children who rotated still paid a time cost with the act of rotation itself. That is, the offloading benefit from rotation was restricted to reduced cognitive demand rather than improved task accuracy or duration.

## 4 Discussion

We adapted Armitage et al.'s (2020) external normalization paradigm to explore whether compelling 6‐ to 9‐year‐old to use an inefficient cognitive offloading strategy influences their subsequent strategy selection. Children in the rotator condition were required to use external normalization across all phase 1 trials, even when doing so did not offload mental rotation demand, whereas children in the non‐rotator condition were prohibited from using external normalization on any phase 1 trial, even when doing so would have offloaded mental rotation demand. When given free strategy choice in phase 2, children in both conditions became more selective in their cognitive offloading with increasing age, using external normalization more often when useful than when redundant. This developmental trajectory is likely underpinned by age‐related improvements in metacognition (Armitage & Gilbert, 2025), as children become increasingly aware of their cognitive struggles (Bayard, van Loon, Steiner, & Roebers, 2021; Roebers & Spiess, 2016; Schneider, Tibken, & Richter, 2022). The findings also align with Armitage et al. (2020) and other relevant work showing improved selectivity and flexibility in strategy use with increasing age (Armitage & Redshaw, 2022; Armitage et al., 2023; Bulley et al., 2020; Redshaw et al., 2018).

Interestingly, older children appeared to become more selective in their offloading decisions after pre‐exposure to an indiscriminate cognitive offloading strategy. Among 8‐ to 9‐year‐olds, those in the phase 1 rotator condition were significantly more selective in their phase 2 cognitive offloading choices than those in the phase 1 non‐rotator condition, with no difference in selectivity between conditions for 6‐ to 7‐year‐olds. One possible explanation is that direct experience with external normalization in the rotator condition in phase 1 made older children more aware of the varying usefulness of the strategy across dimensions, which instead had to be inferred by children in the non‐rotator condition. This interpretation aligns with foundational theories of constructivism, which emphasize the importance of active involvement and participation for children's learning and development (e.g., Piaget, 1973). Indeed, given the well‐established developmental delay between metacognitive knowledge and control (see Schneider et al., 2008, for a review), it is possible that even the younger children in the rotator condition came to recognize the differential usefulness of the offloading technique in phase 1, yet they simply lacked the ability to translate such knowledge into more selective offloading when given the opportunity in phase 2.

Promisingly, however, neither younger nor older children who were compelled to offload in phase 1 showed any sign of increased redundancy in their phase 2 offloading strategies, in that they did not show an increased propensity to rotate the turntable when counting based on the color dimension. Notably, this contrasts with previous work showing that adults who have been pre‐exposed to excessive offloading strategies continue to persevere with these suboptimal strategies even when given a free choice (Scarampi & Gilbert, 2020) or an easier task (Storm et al., 2016). One key difference, however, is that in our task, there was no benefit at all afforded by manual rotation on color trials, such that the inefficient strategy involved a completely redundant behavior. By contrast, adults’ strategy perseverance effects were observed in trials where offloading was *less useful* (i.e., when trying to recall one rather than five intended actions, as in Scarampi & Gilbert, 2020; or when answering easy compared to difficult trivia questions, as in Storm et al., 2016), but not entirely redundant. Future work should, therefore, explore whether children's offloading strategy perseveration tendencies differ in contexts where the available strategy has varying levels of usefulness.

At face value, the finding that children did not carelessly deploy the offloading technique after being first taught to routinely use it may be a positive sign amid concerns about long‐term impacts of excessive offloading on children's thinking and behavior (Armitage & Gilbert, 2025). However, our accuracy and time‐on‐task analyses gave rise to some unexpected findings—most notably that the benefit afforded by rotating the turntable when counting arms appeared limited to reduced cognitive demand, rather than an improvement in counting accuracy or overall task duration. While there is some evidence that offloading cognitive demand helps children free up cognitive resources to be allocated elsewhere (e.g., Goldin‐Meadow, Nusbaum, Kelly, & Wagner, 2001), our task did not include any concurrent demands in which such cognitive savings could be reinvested. An alternative interpretation of our results is, therefore, that children's selectivity was motivated by a desire to think less rather than perform better. Given that receptiveness to cognitive stimulation promotes cognitive development across a range of domains (Rakesh, McLaughlin, Sheridan, Humphreys, & Rosen, 2024), this interpretation raises important questions about whether children *should* be offloading merely to reduce cognitive demand, or whether this behavior is better reserved for contexts in which it yields overall performance benefits.

Furthermore, our findings can only speak to possible impacts of short‐term pre‐exposure on children's selective offloading decisions. It remains possible that repeated pre‐exposure over a longer duration of time has more pronounced or enduring effects on children's subsequent strategy selection, including their tendency to offload with improved overall task performance in mind. To further our understanding of children's responses to strategy pre‐exposure, future work should explore the influence of longer exposure durations, as well as the generalizability of our findings across non‐WEIRD child samples (Henrich, Heine, & Norenzayan, 2010) and diverse cognitive domains (Armitage & Gilbert, 2025). Nonetheless, here we have provided the first evidence that, in some cases at least, children become more selective in their cognitive offloading decisions following pre‐exposure to excessive cognitive offloading strategies.

## Author contributions

K.L.A. assisted with study design, analyzed the data, wrote the first draft, and edited the manuscript. A.K.J. assisted with study design, collected data, and edited the manuscript. J.R. assisted with study design, supervised the project, and edited the manuscript.

## Funding information

This work was supported by an ARC Discovery Early Career Researcher Award (DE210100005), ARC Future Fellowship (FT230100010), and ARC Discovery Project grant (DP250102648) awarded to J.R., and an ARC Discovery Project grant (DP250100833) awarded to K.L.A. It was also supported by a James S. McDonnell Foundation Opportunity Award granted to J.R. (2022‐3781).

## Conflicts of Interest Statement

The authors hereby declare no conflicts of interest.

## Ethical approval statement

Ethical approval was obtained through the University of Queensland's Ethics Committee (Clearance ID: 2019/HE000267).

## Supporting information



Supporting Information

## Data Availability

The dataset is publicly available through the Open Science Framework repository (https://osf.io/9ghrv/).
