# Supplementary material for: Children Show More Selective Cognitive Offloading After First Being Compelled to Offload Indiscriminately
Source: Cogn Sci. 2025 Aug 5;49(8):e70100. doi: 10.1111/cogs.70100 (PMC12323294; doi:10.1111/cogs.70100)
Supplement: Supplementary file 1 — Supporting Information [file COGS-49-e70100-s001.docx]

Supplementary Materials for:

**Children show more selective cognitive offloading after first being compelled to offload indiscriminately**

Kristy L. Armitage, Alicia K. Jones & Jonathan Redshaw

Published in *Cognitive Science*

**S1 | Additional Activities**

**Counting Screening Measure**

Children were required to pass a screening procedure assessing their ability to count to 10. A square stimulus sheet (28.5cm x 28.5cm) containing 10 green squares and 8 yellow squares (3cm x 3cm) was placed in front of children. Children who counted 10 or more green squares were considered to have passed the screening procedure and progressed to the experimental task. No children failed this screening measure.


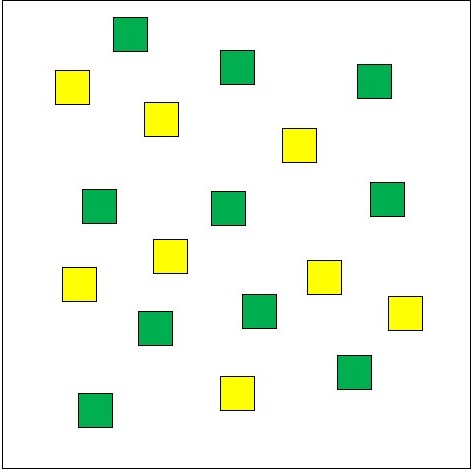


**Preliminary Rotation Activity**

To ensure children understood that the turntable could be used for manual rotation prior to beginning the experimental task, all children completed a preliminary rotation activity (as in Armitage et al., 2020). After being informed that the turntable could rotate, children were presented with a stimulus sheet (28.5cm in diameter) showing four animals. Children were asked to determine which animal was upside down, and then used the turntable to rotate the animal until it was upright. For example, in the below orientation, children would be asked to make the bird upright by rotating the turntable. Children performed this rotation four times (once for each animal). Figure reproduced from Armitage et al. (2020; supplementary materials).


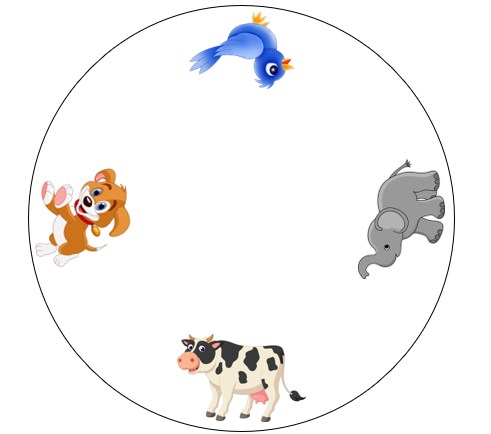


**S2 | Experimenter Script**

*Experimenter actions are in italics.*

**Before Phase 1**

*Place the counting screening measure on the floor in front of the child.* Can you please count the green squares for me?

*Place an example stimulus sheet on the floor in front of the child.* Now I want you to look at this picture here. Can you see that there are blue people and red people? And can you see that there are people with their arms pointing up and people with their arms pointing down? Some of the blue people have their arms pointed up, and some have their arms pointed down. Some of the red people have their arms pointed up, and some have their arms pointed down. Can you point to a blue person with their arms up? Can you point to a blue person with their arms down? Can you point to a red person with their arms up? Can you point to a red person with their arms down?

*Place the preliminary rotation activity on the turntable in front of the child.* This is a rotating turntable; we can spin this around. Do you see these animals? Some of these animals are upside down. Can you make this animal the right way up? *Repeat 4 times (once for each animal).*

**Phase 1**

For this activity, I’m going to put some of these sheets onto the top of this table. I’m going to place the sheets upside down like this. *Demonstrate with an example stimulus sheet.*

Rotator Condition: I want you to count some things, but first you have to make the sheets the right way up, okay?

Non-Rotator Condition: I want you to count some things, but you have to leave the sheets upside down, okay?

**Phase 2**

This time, we’re going to play the same game, but you have a choice. Do you know what a choice is? It’s when you get to decide what to do. I’m going to place the sheets upside down again like this. *Demonstrate with an example stimulus sheet.* This time, you can choose if you want to turn the table to make the people the right way up for you, like this. *Demonstrate rotating the turntable.* You can move this table as much as you want to. Whenever you want to move it, you can. But you don’t have to – it’s your choice.

**S3 | Counterbalancing**

Sixteen stimulus sheets (28.5cm in diameter) were created and laminated, with Velcro used to attach each sheet to the turntable (39cm in diameter). Each sheet featured a unique combination of red and blue stick figures, with their arms pointing either up or down. Children saw each sheet once during the experiment, with the number of blue figures and figures with their arms up ranging from 6 to 10. Importantly, the number of blue figures and figures with their arms up were different on all sheets, eliminating the possibility of easy shortcuts, like counting the blue figures on more difficult trials where children should be counting the figures with their arms up.

Children completed 8 trials in phase 1 (4 colour, 4 arms) and 8 trials in phase 2 (4 colour, 4 arms) in one of four possible orders:

|  | Phase 1 | | Phase 2 | |
| --- | --- | --- | --- | --- |
| Order | Trial Type | Stimulus Sheets | Trial Type | Stimulus Sheets |
| 1 | Arms, Arms, Colour, Colour, Arms, Arms, Colour, Colour | 1 - 8 | Arms, Arms, Colour, Colour, Arms, Arms, Colour, Colour | 9 - 16 |
| 2 | Colour, Colour, Arms, Arms, Colour, Colour, Arms, Arms | 1 - 8 | Colour, Colour, Arms, Arms, Colour, Colour, Arms, Arms | 9 - 16 |
| 3 | Arms, Arms, Colour, Colour, Arms, Arms, Colour, Colour | 16 - 9 | Arms, Arms, Colour, Colour, Arms, Arms, Colour, Colour | 8 - 1 |
| 4 | Colour, Colour, Arms, Arms, Colour, Colour, Arms, Arms | 16 - 9 | Colour, Colour, Arms, Arms, Colour, Colour, Arms, Arms | 8 - 1 |

The same orders were used for each condition (rotator, non-rotator), creating a total of 8 possible counterbalancing conditions. An equal number of children in each age group (n = 4) completed each counterbalancing condition.

**S4 | Phase 1 Accuracy LMMs**

The following models were preregistered without the focal effect of trial or the control variables of sex and counterbalancing order.

| Effects | *df* | $\chi^{2}$ | *p* | *w* |
| --- | --- | --- | --- | --- |
| *Base model* (-2LL = 2379.26) |  |  |  |  |
| Intercept (PID): *z* = 4.12, *p* < .001 |  |  |  |  |
| Sex (control) | 1, 893 | 1.50 | .222 | -0.11 |
| Order (control) | 1, 893 | 0.22 | .637 | -0.04 |
| Trial | 1, 893 | 2.07 | .150 | -0.13 |
| **Age** | **1, 893** | **20.21** | **< .001** | -0.40 |
| Condition | 1, 893 | 1.46 | .227 | -0.11 |
| **Dimension** | **1, 893** | **250.66** | **< .001** | 1.40 |
| *Interaction model 1* (-2LL = 2376.39) |  |  |  |  |
| Intercept (PID): *z* = 4.13, *p* < .001 |  |  |  |  |
| Condition x Dimension | 1, 892 | 2.88 | .090 | 0.15 |
| *Interaction model 2* (-2LL = 2358.20) |  |  |  |  |
| Intercept (PID): *z* = 4.21, *p* < .001 |  |  |  |  |
| **Age x Dimension** | **1, 892** | **21.31** | **< .001** | **0.41** |
| *Interaction model 3* (-2LL = 2355.54) |  |  |  |  |
| Intercept (PID): *z* = 4.23, *p* < .001 |  |  |  |  |
| **Age x Dimension x Condition** | **3, 891** | **21.32** | **< .001** | **0.41** |

The base model outperformed interaction model 1, $\chi^{2}$(1) = 2.87, *p* = .090, but was outperformed by interaction model 2, $\chi^{2}$(1) = 21.06, *p* < .001, and interaction model 3, $\chi^{2}$(4) = 23.72, *p* < .001, in Likelihood Ratio Tests.

Interaction model 2 follow-up

|  | *df* | $\chi^{2}$ | *p* | *w* |
| --- | --- | --- | --- | --- |
| **Effect of dimension, younger children** | **1, 893** | **194.26** | **<. 001** | **1.74** |
| **Effect of dimension, older children** | **1, 893** | **74.44** | **< .001** | **1.08** |

*Note.* Bonferroni corrections applied.

Interaction model 3 follow-up

|  | *df* | $\chi^{2}$ | *p* | *w* |
| --- | --- | --- | --- | --- |
| Effect of condition, colour dimension, younger children | 1, 891 | 0.02 | > .999 | 0.02 |
| Effect of condition, colour dimension, older children | 1, 891 | 0.06 | > .999 | -0.03 |
| Effect of condition, arms dimension, younger children | 1, 891 | 1.24 | > .999 | -0.14 |
| Effect of condition, arms dimension, older children | 1, 891 | 2.72 | .397 | -0.21 |

*Note.* Bonferroni corrections applied.

**S5 | Phase 2 Rotation GLMMs (Preregistered)**

As specified in the Method, children received permission to rotate the turntable after every second phase 2 trial: “Remember, you can move it if you want to, but you don’t have to”. This was included to mitigate issues with perceived permission, especially for children in the non-rotator condition who were unable to rotate in phase 1. We ran an exploratory GLMM, modelling phase 2 rotation as a function of age, condition, dimension, trial, and reminder (binary; 1 = trials immediately following a reminder, 0 = all other trials). A significant main effect of reminder, χ2(1, 892) = 15.66, p < .001, w = 0.35, indicated that children rotated the turntable significantly more frequently on trials immediately following a reminder. This did not significantly interact with age, χ2(1, 891) = 1.40, p = .237, w = 0.10, dimension, χ2(1, 892) = 3.42, p = .064, w = 0.16, or condition, χ2(1, 892) = 2.65, p = .103, w = 0.14. Based on these results, we now include this reminder variable as a control variable in all analyses involving the phase 2 rotation DV.

The following models were preregistered without the control variables of sex, counterbalancing order, and reminder.

| Effects | *df* | $\chi^{2}$ | *p* | *w* |
| --- | --- | --- | --- | --- |
| *Base model* (-2LL = 945.31) |  |  |  |  |
| Intercept (PID): *z* = 4.58, *p* < .001 |  |  |  |  |
| Sex (control) | 1, 892 | 0.95 | .329 | 0.07 |
| Order (control) | 1, 892 | 0.14 | .711 | 0.03 |
| Reminder (control) | 1, 892 | 15.66 | < .001 | 0.35 |
| Condition | 1, 892 | 2.60 | .107 | 0.14 |
| **Dimension** | **1, 892** | **210.07** | **< .001** | **1.28** |
| Trial | 1, 892 | 0.53 | .467 | -0.06 |
| Age | 1, 892 | 3.82 | .051 | -0.17 |
| *Interaction model 1* (-2LL = 942.46) |  |  |  |  |
| Intercept (PID): *z* = 4.56, *p* < .001 |  |  |  |  |
| Condition x Dimension | 1, 891 | 2.85 | .092 | 0.15 |
| *Interaction model 2* (-2LL = 945.02) |  |  |  |  |
| Intercept (PID): *z* = 4.58, *p* < .001 |  |  |  |  |
| Condition x Trial | 1, 892 | 0.29 | .590 | 0.05 |
| *Interaction model 3* (-2LL = 937.94) |  |  |  |  |
| Intercept (PID): *z* = 4.58, *p* < .001 |  |  |  |  |
| **Dimension x Trial** | **1, 892** | **7.23** | **.007** | **0.24** |
| *Interaction model 4* (-2LL = 934.25) |  |  |  |  |
| Intercept (PID): z = 4.57, *p* < .001 |  |  |  |  |
| Dimension x Trial x Condition | 2, 889 | 3.67 | .160 | 0.17 |
| *Interaction model 5* (-2LL = 919.77) |  |  |  |  |
| Intercept (PID): z = 4.52, *p* < .001 |  |  |  |  |
| **Dimension x Age** | **1, 892** | **22.69** | **< .001** | **0.42** |
| *Interaction model 6* (-2LL = 943.10) |  |  |  |  |
| Intercept (PID): z = 4.55, *p* < .001 |  |  |  |  |
| Condition x Age | 1, 893 | 2.22 | .137 | 0.13 |
| Interaction model 7 (-2LL = 912.08) |  |  |  |  |
| Intercept (PID): z = 4.45, *p* < .001 |  |  |  |  |
| **Condition x Age x Dimension** | **3, 890** | **26.20** | **< .001** | **0.45** |

The base model was outperformed by interaction model 3, $\chi^{2}$(1) = 7.37, *p* = .007, interaction model 4, $\chi^{2}$(4) = 11.06, *p* = .026, interaction model 5, $\chi^{2}$(1) = 25.54, *p* < .001, and interaction model 7, $\chi^{2}$(4) = 33.23, *p* < .001, but no other interaction models, $\chi^{2}$s < 2.85, *p*s > .091, in Likelihood Ratio Tests.

Interaction model 3 follow-up

|  | *df* | $\chi^{2}$ | *p* | *w* |
| --- | --- | --- | --- | --- |
| Trial effect, colour dimension | 1, 892 | 2.03 | .308 | -0.13 |
| Trial effect, arms dimension | 1, 892 | 5.00 | .051 | 0.20 |

*Note.* Bonferroni corrections applied.

Interaction model 5 follow-up

|  | *df* | $\chi^{2}$ | *p* | *w* |
| --- | --- | --- | --- | --- |
| Dimension effect, younger children | 1, 892 | 98.85 | < .001 | 1.24 |
| Dimension effect, older children | 1, 892 | 137.15 | < .001 | 1.46 |

*Note.* Bonferroni corrections applied.

Interaction model 7 follow-up

|  | *df* | $\chi^{2}$ | *p* | *w* |
| --- | --- | --- | --- | --- |
| Condition effect, colour dimension, younger children | 1, 890 | 0.15 | > .999 | 0.05 |
| Condition effect, colour dimension, older children | 1, 890 | 0.01 | > .999 | -0.01 |
| Condition effect, arms dimension, younger children | 1, 890 | 0.00 | > .999 | - <0.01 |
| Condition effect, arms dimension, older children | 1, 890 | 10.45 | .005 | 0.40 |

*Note.* Bonferroni corrections applied.

**S6 | Binomial Regressions**

Following Armitage et al. (2020), children were organised into one of three mutually exclusive categories based on their rotation behaviours in phase 2: (i) *selective* *rotation*, where children rotated the turntable on at least one arms trial and no colour trials, (ii) *indiscriminate* *rotation*, where children rotated the turntable on at least one trial, including at least one colour trial, and (iii) *no rotation,* where children did not rotate the turntable on any trial (see the figure below). Note that no reliability coding was carried out for this categorization process given that it (a) involved minimal ambiguity and could be calculated using an existing variable (rotation) and (b) does not appear in the main text.

As preregistered, a logistic regression examined whether selective rotation (1 = selective rotation, 0 = indiscriminate or no rotation) varied as a function of age (continuous, mean-centred) and condition (rotator or non-rotator). In step one the main effect of age was significant, *Wald χ*^2^(1, *N* = 128) = 12.88, *p* < .001, *w* = 0.32 (b = 0.62), confirming that children were more likely to be selective rotators with increasing age. The main effect of condition was non-significant, *Wald χ*^2^(1, *N* = 128) = 1.65, *p* = .199, *w* = 0.11 (b = 0.49), indicating that pre-exposure to excessive external normalisation did not impact children’s likelihood of being classified as a selective rotator. In step two, there was no evidence of an interaction between age and condition, *Wald χ*^2^(1, *N* = 128) < 0.01, *p* = .979, *w* < 0.01 (b = 0.01). The same pattern of results was found when excluding non-rotators from the analysis.


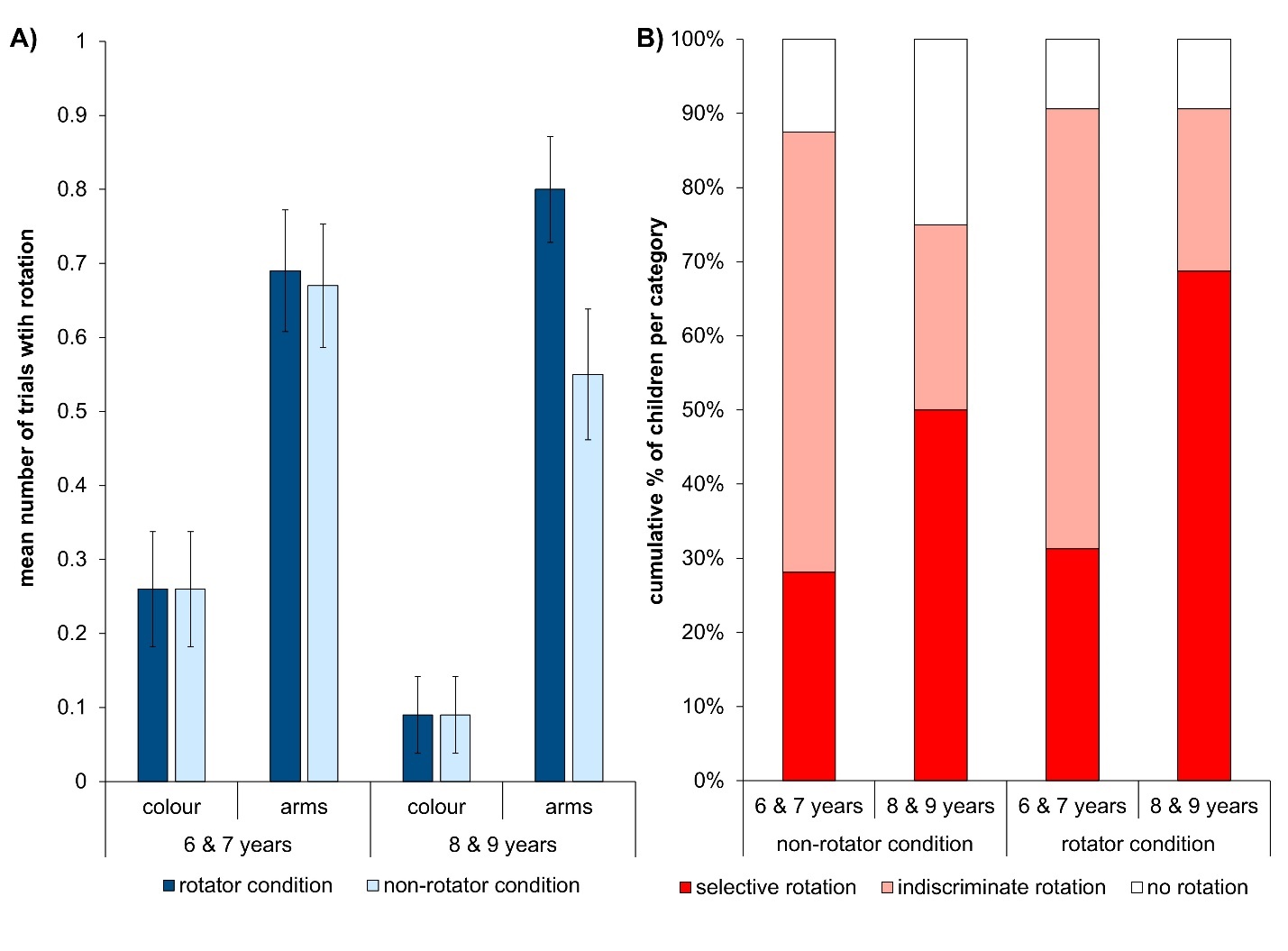


**S7 | Phase 2 Accuracy GEEs**

See the Analysis Plan in the main text for information about the distribution of residuals for the Phase 2 Accuracy DV. The following models were preregistered without the focal effect of trial or the control variables of sex and counterbalancing order.

| Effects | *df* | $\chi^{2}$ | *p* | *w* |
| --- | --- | --- | --- | --- |
| *Base model* |  |  |  |  |
| Sex (control) | 1 | 0.92 | .336 | -0.08 |
| Order (control) | 1 | 0.00 | .979 | < 0.01 |
| Trial | 1 | 0.97 | .324 | -0.09 |
| **Age** | **1** | **12.72** | **< .001** | -0.32 |
| Condition | 1 | 0.06 | .802 | -0.02 |
| **Dimension** | **1** | **42.20** | **< .001** | **0.57** |
| Rotation | 1 | 0.02 | .897 | 0.01 |
| *Interaction model 1* |  |  |  |  |
| Dimension x Rotation | 1 | 1.06 | .302 | 0.09 |
| *Interaction model 2* |  |  |  |  |
| **Age x Dimension (Exploratory)** | **1** | **9.43** | **.002** | **0.27** |
| *Interaction model 3* |  |  |  |  |
| **Dimension x Rotation x Age (Exploratory)** | **3** | **11.51** | **.009** | **0.30** |

Interaction model 2 follow-up

|  | *df* | $\chi^{2}$ | *p* | *w* |
| --- | --- | --- | --- | --- |
| **Dimension effect, younger children** | **1** | **59.93** | **< .001** | **0.97** |
| **Dimension effect, older children** | **1** | **28.83** | **< .001** | **0.67** |

*Note.* Bonferroni corrections applied.

Interaction model 3 follow-up

|  | *df* | $\chi^{2}$ | *p* | *w* |
| --- | --- | --- | --- | --- |
| Rotation effect for colour trials, younger children | 1 | < 0.01 | > .999 | < 0.01 |
| Rotation effect for colour trials, older children | 1 | < 0.01 | > .999 | < 0.01 |
| Rotation effect for arms trials, younger children | 1 | 0.37 | > .999 | 0.08 |
| Rotation effect for arms trials, older children | 1 | < 0.01 | > .999 | < 0.01 |

*Note.* Bonferroni corrections applied.

**S8 | Time-on-Task Analyses (Exploratory)**

**Phase 1**

In phase 1, time-on-task was measured either from the moment the stimulus sheet was placed on the turntable (for non-rotators) or was rotated to the upright orientation (for rotators) to the moment the child provided an answer (to reflect the period that children should have been counting in each condition).

**Phase 1 Time-on-Task GLM**

| Effects | *df* | $\chi^{2}$ | *p* | *w* |
| --- | --- | --- | --- | --- |
| *Base model (-2LL = 4766.71)*  Intercept (PID): z = 4.51, *p* < .001 |  |  |  |  |
| Sex (control) | 1, 672 | 0.01 | .922 | 0.01 |
| Order (control) | 1, 672 | 0.05 | .816 | 0.02 |
| **Trial** | **1, 672** | **26.07** | **< .001** | **-0.45** |
| **Age** | **1, 672** | **6.89** | **.009** | **-0.23** |
| **Condition** | **1, 672** | **8.62** | **.003** | **-0.26** |
| **Dimension** | **1, 672** | **1111.43** | **< .001** | **2.95** |
| *Interaction model 1 (-2LL = 4743.14)*  Intercept (PID): z = 4.59, *p* < .001 |  |  |  |  |
| **Dimension x Condition** | **1, 671** | **24.76** | **< .001** | **0.44** |
| *Interaction model 2 (-2LL = 4764.35)*  Intercept (PID): z = 4.52, *p* < .001 |  |  |  |  |
| Age x Dimension | 1, 671 | 2.37 | .124 | 0.14 |
| *Interaction model 3 (-2LL = 4759.86)*  Intercept (PID): z = 4.53, *p* < .001 |  |  |  |  |
| Age x Dimension x Condition | 2, 670 | 4.46 | .107 | 0.19 |

The base model was outperformed by interaction model 1, $\chi^{2}$(1) = 23.57, *p* < .001, but no other interaction models, $\chi^{2}$s < 6.85, *p*s > .077.

Interaction model 1 follow-up

|  | *df* | $\chi^{2}$ | *p* | *w* |
| --- | --- | --- | --- | --- |
| Condition effect, colour trials | 1, 671 | 0.01 | > .999 | 0.01 |
| **Condition effect, arms trials** | **1, 671** | **25.01** | **< .001** | **0.44** |

*Note.* Bonferroni corrections applied.

**Phase 2 Time-on-Task GEE**

In phase 2, time-on-task was measured from the moment the stimulus sheet was placed on the turntable to the moment children provided an answer (given that all children had a choice to rotate or not prior to counting).

See the Analysis Plan in the main text for information about the distribution of residuals for the Phase 2 Time-on-Task DV.

| Effects | *df* | $\chi^{2}$ | *p* | *w* |
| --- | --- | --- | --- | --- |
| *Base model* |  |  |  |  |
| Sex (control) | 1 | < 0.01 | .982 | < 0.01 |
| Order (control) | 1 | 0.02 | .891 | 0.01 |
| Trial | 1 | 0.60 | .438 | -0.07 |
| **Age** | 1 | **10.52** | **.001** | **-0.29** |
| Condition | 1 | 0.49 | .483 | -0.06 |
| **Dimension** | 1 | **62.26** | **< .001** | **0.70** |
| Rotation | 1 | 2.93 | .087 | 0.15 |
| *Interaction model 1* |  |  |  |  |
| **Dimension x Rotation** | **1** | **6.56** | **.010** | **0.23** |
| *Interaction model 2* |  |  |  |  |
| Age x Dimension | 1 | 2.67 | .102 | 0.14 |
| *Interaction model 3* |  |  |  |  |
| Age x Dimension x Rotation | 3 | 3.12 | .374 | 0.16 |

Interaction model 1 follow-up

|  | *df* | $\chi^{2}$ | *p* | *w* |
| --- | --- | --- | --- | --- |
| **Rotation effect, colour trials** | **1** | **27.34** | **< .001** | **0.46** |
| Rotation effect, arms trials | 1 | < 0.01 | > .999 | < 0.01 |

*Note.* Bonferroni corrections applied.

**S9 | Binomial Accuracy Analyses (Exploratory)**

We ran our phase 1 and phase 2 accuracy analyses twice – once with a continuous accuracy DV (difference score between given answer and correct answer, as preregistered and reported in text) and again with a binomial accuracy DV (correct or incorrect; exploratory). The pattern of results for both phases was consistent across both DVs.

**Phase 1 Accuracy (GLMM – binomial DV)**

| Effects | *df* | $\chi^{2}$ | *p* | *w* |
| --- | --- | --- | --- | --- |
| *Base model* |  |  |  |  |
| Sex (control) | 1, 893 | 3.77 | .052 | 0.17 |
| Order (control) | 1, 893 | 0.06 | .804 | 0.02 |
| Trial | 1, 893 | 1.98 | .159 | 0.12 |
| **Age** | **1, 893** | **16.35** | **< .001** | 0.36 |
| Condition | 1, 893 | 0.82 | .364 | 0.08 |
| **Dimension** | **1, 893** | **308.86** | **< .001** | -1.55 |
| *Interaction model 1* |  |  |  |  |
| Condition x Dimension | 1, 892 | 2.76 | .097 | 0.15 |
| *Interaction model 2* |  |  |  |  |
| **Age x Dimension** | **1, 892** | **10.74** | **.001** | **0.29** |
| *Interaction model 3* |  |  |  |  |
| **Age x Dimension x Condition** | **3, 891** | **11.55** | **.009** | **0.30** |

Interaction model 2 follow-up

|  | *df* | $\chi^{2}$ | *p* | *w* |
| --- | --- | --- | --- | --- |
| **Effect of dimension, younger children** | **1, 893** | **199.11** | **<. 001** | **1.76** |
| **Effect of dimension, older children** | **1, 893** | **117.03** | **< .001** | **1.35** |

*Note.* Bonferroni corrections applied.

Interaction model 3 follow-up

|  | *df* | $\chi^{2}$ | *p* | *w* |
| --- | --- | --- | --- | --- |
| Effect of condition, colour dimension, younger children | 1, 891 | 0.14 | > .999 | -0.05 |
| Effect of condition, colour dimension, older children | 1, 891 | 0.02 | > .999 | 0.02 |
| Effect of condition, arms dimension, younger children | 1, 891 | 2.92 | .350 | 0.21 |
| Effect of condition, arms dimension, older children | 1, 891 | 0.45 | > .999 | 0.08 |

*Note.* Bonferroni corrections applied.

**S10 | Effect of Phase 1 Accuracy on Phase 2 Rotation (Exploratory)**

We ran exploratory analyses examining whether children’s phase 1 accuracy influenced their phase 2 rotation. A “phase 1 accuracy on trial type” variable was calculated for each child by averaging their accuracy scores (continuous) for each dimension in phase 1. We then modelled children’s use of rotation in phase 2 as a function of age, condition, dimension, trial and phase 1 accuracy on trial type, controlling for sex, counterbalancing order, and reminders (see S5 in the supplementary materials for an overview of the ‘reminders’ variable).

The main effect of phase 1 accuracy on trial type was non-significant, but this significantly varied in a two-way interaction with age, and a three-way interaction with age and condition. However, all follow-up analyses were non-significant, suggesting that the significant interaction terms likely represented a significant shift in the direction or strength of the accuracy effect as a function of the other variables, with no individual effects reaching conventional significance. All follow-up effects indicated a negative relationship between phase 1 accuracy on trial type and phase 2 rotation, which was stronger for older children than younger children (see interaction model 3 follow-up), especially those in the rotator condition (see interaction model 4 follow-up). In other words, children with *lower* difference scores in phase 1 (i.e., higher accuracy) showed higher rates of offloading in phase 2. These findings align with Armitage & Redshaw (2022), where children only began to use cognitive offloading to compensate for poorer unaided performance around age 10 and 11 years.

| Effects | *df* | $\chi^{2}$ | *p* | *w* |
| --- | --- | --- | --- | --- |
| *Base model* |  |  |  |  |
| Sex (control) | 1, 891 | 0.84 | .360 | 0.08 |
| Order (control) | 1, 891 | 0.13 | .717 | 0.03 |
| **Reminder (control)** | **1, 891** | **15.70** | **< .001** | **0.35** |
| Condition | 1, 891 | 2.43 | .119 | 0.14 |
| Dimension | 1, 891 | **133.36** | **< .001** | **1.02** |
| Trial | 1, 891 | 0.53 | .467 | -0.06 |
| **Age** | **1, 891** | **4.07** | **.044** | **-0.18** |
| Phase 1 Accuracy on Trial Type | 1, 891 | 0.34 | .560 | -0.05 |
| *Interaction model 1* |  |  |  |  |
| Phase 1 Accuracy on Trial Type x Condition | 1, 891 | 0.06 | .804 | 0.02 |
| *Interaction model 2* |  |  |  |  |
| Phase 1 Accuracy on Trial Type x Dimension | 1, 891 | 1.48 | .225 | 0.11 |
| *Interaction model 3* |  |  |  |  |
| **Phase 1 Accuracy on Trial Type x Age** | **1, 890** | **5.93** | **.015** | **0.22** |
| *Interaction model 4* |  |  |  |  |
| **Phase 1 Accuracy on Trial Type x Condition x Age** | **2, 889** | **7.87** | **.020** | **0.25** |

Interaction model 3 follow-up

|  | *df* | $\chi^{2}$ | *p* | *w* |
| --- | --- | --- | --- | --- |
| Accuracy effect, younger children | 1, 891 | 1.33 | .499 | -0.14 |
| Accuracy effect, older children | 1, 891 | 3.22 | .145 | -0.22 |

*Note.* Bonferroni corrections applied.

Interaction model 4 follow-up

|  | *df* | $\chi^{2}$ | *p* | *w* |
| --- | --- | --- | --- | --- |
| Accuracy effect, non-rotator, younger children | 1, 889 | 0.30 | >.999 | -0.07 |
| Accuracy effect, non-rotator, older children | 1, 889 | 0.37 | >.999 | -0.08 |
| Accuracy effect, rotator, younger children | 1, 889 | 1.05 | >.999 | -0.13 |
| Accuracy effect, rotator, older children | 1, 889 | 5.61 | .072 | -0.30 |

*Note.* Bonferroni corrections applied.
